# Patient Perceptions about Professional Dental Services during the COVID-19 Pandemic

**DOI:** 10.1177/2380084420969116

**Published:** 2020-10-21

**Authors:** R.C. Moffat, C.T. Yentes, B.T. Crookston, J.H. West

**Affiliations:** 1 College of Dental Medicine, Roseman University, South Jordan, UT, USA; 2 Department of Public Health, Brigham Young University, Provo, UT, USA

**Keywords:** psychosocial factors, access to care, clinical practice guidelines, community dentistry, dental health survey, dental public health

## Abstract

**Background:**

Dental professionals are at high risk of being infected by and transmitting COVID-19 to patients. Patients’ perceived risk for infection and attitudes about receiving dental care during the pandemic are important to understand as patients consider returning to routine dental care as the pandemic progresses.

**Objective:**

The purpose of this study was to explore dental patients’ perceptions of susceptibility to contracting COVID-19, their related attitudes and beliefs regarding dental care visits, and their considerations for returning to routine care during and after the pandemic.

**Method:**

Data for this cross-sectional study came from an electronic survey of 464 US adults. Survey variables include demographics, dental hygiene behaviors, perceived susceptibility to COVID-19, attitudes and beliefs regarding risk for attending dental appointments, and the necessary conditions and events for them to feel comfortable returning to regular dental appointments.

**Results:**

Over half of study participants had a 4-y degree, an annual income of at least $50,000, and good oral hygiene practices of frequent brushing and routine dental visits. Older age and agreement with positive attitudinal statements and beliefs about professional dental care were positively related to perceived susceptibility for contracting COVID-19 in a dental setting. Perceptions of susceptibility, a higher valuation of dentistry, and agreement that COVID-19 is a serious infection were each positively related to attitudinal statements and beliefs reflecting caution in attending dental visits. Last, assurance from public health officials confirming the safety to return for routine dental care was the largest reported factor necessary for a return to routine dental visits.

**Conclusion:**

This study provides early data about patient perceptions of susceptibility and attitudes toward COVID-19 in a professional dental setting and necessary conditions for returning to regular visits. This information can help formulate messaging related to returning to professional dental care, specifically targeting fears among the most susceptible populations.

**Knowledge Transfer Statement:**

Government and public health agencies can play an important role in alleviating concerns and instilling confidence that dental settings are safe. With this information from the public, dental professionals and public health agencies can work together to share messaging that will consistently inform the public regarding the safety of returning to professional dental care as it relates to the reopening of states and cities.

## Introduction

The novel coronavirus (SARS-CoV-2) pandemic is a highly disruptive event that has significantly affected daily life in nearly every country. Individuals infected with COVID-19, the disease caused by SARS-CoV-2, are found in >200 countries and in all 50 of the United States. Millions of people have been infected worldwide (Johns Hopkins University and Medicine 2020). COVID-19 is highly contagious, with a rapid velocity of transmission, and can be spread by symptomatic and asymptomatic carriers. The modes of transmission include droplets, surface contact, and aerosolization (Centers for Disease Control and Prevention [CDC] 2020a).

Clinicians interacting with multiple patients can serve as vectors for viral transmission between patients (Chen et al. 2020). Due to close physical contact with patients, dental professionals are at particularly high risk of being infected by, as well as transmitting COVID-19 to, their patients (Meng et al. 2020). In Italy, several dental professionals died of COVID-19 as early as the initial stages of the pandemic (Chustecka 2020). Currently, the best protection for dental professionals and their patients is increasing awareness, avoiding unnecessary contact with people who may have COVID-19, using appropriate personal protective equipment, and increasing attention to engaging in personal hygiene behaviors, especially handwashing (CDC 2020b). Because of the high risk for dental professionals and their patients of contracting COVID-19, on March 16, 2020, the American Dental Association (2020) recommended that dental professionals postpone elective procedures and provide only emergency dental care. Additionally, the CDC (2020b) recommended that dental health care providers delay all elective provider visits and suspend routine dental visits. At the time of writing, although routine dental visits have resumed in the United States, it is unclear when or to what extent clinic operations will fully return to the pre–COVID-19 norm.

Managing the balance between addressing patient concerns and safety related to COVID-19 transmission and upholding long-standing practices of regular visits for prevention and treatment presents a new challenge to dental health. The health belief model (HBM) provides a lens through which to explore the relationship between the COVID-19 outbreak and patients’ perceptions of susceptibility as well as their attitudes and beliefs. In the HBM, if individuals perceive themselves as being susceptible to a pathologic condition and believe that the condition would have potentially serious consequences, then they may be more inclined to adopt prohealth attitudes and health behaviors to address this condition (Champion and Skinner 2008). For example, they may believe that removing themselves from scenarios that may increase risk of exposure, as in a dental office, will be beneficial in avoiding COVID-19. As such, they may be more inclined to cancel dental appointments, maybe even in cases of an emergency. Little is known about the beliefs, attitudes, and perceptions that dental patients have regarding susceptibility to being infected with COVID-19 as a result of professional dental care during this pandemic.

The purpose of this study was to explore the perceptions and the construct of attitudes and beliefs of dental patients regarding professional dental care early in the COVID-19 pandemic. A secondary purpose was to determine the conditions and events that will influence dental patients to return to pre–COVID-19 dental appointment routines. This study will serve to guide dental and public health professionals and patients as they seek to reestablish positive dental health care practices in the midst of the COVID-19 pandemic.

## Methods

### Design and Sample

Data for this study came from a cross-sectional survey. The study sample comprised respondents who were recruited through Amazon Mechanical Turk (MTurk). MTurk is a crowdsourcing Internet marketplace that enables organizations to coordinate the completion of tasks, of which surveys are a common variety. MTurk prequalifies potential participants who express interest in participation after seeing a posting on MTurk that described the survey. The description was as follows: “Complete a 20-minute survey about your beliefs, attitudes, and perceptions regarding professional dental care during the COVID-19 pandemic.” The sample was limited to respondents who were ≥18 y old and residents of the United States. A total of 464 respondents completed the survey. The Institutional Review Board of Brigham Young University approved this study.

### Procedure

An electronic survey was created in Qualtrics and then linked to MTurk. The first item on the survey was informed consent. The entire survey took approximately 20 min to complete, and participants received $3 for their participation. The survey was developed in April 2020 and pilot-tested with a small group of health professionals, including a physician, a dentist, and a nurse. Pilot-test participants were asked to provide feedback on the understandability of specific items and general survey flow. Updates to the survey were made according to the feedback in the pilot test, and the final survey was administered in early May 2020. This was approximately 1.5 mo after the implementation of federal and state guidelines to limit social gathering and the American Dental Association recommendations for dental offices to perform only emergency procedures.

### Measurement

The HBM was used to inform the development of the survey items. HBM emphasizes the relationship between perceptions of susceptibility for infection and severity of the ensuing illness, both of which are presumed to influence individuals’ risk avoidance behaviors. Participants were asked to respond to demographic items, including age, gender, income, race/ethnicity, and general oral hygiene behaviors (i.e., frequency of brushing and flossing, consistency of routine dental appointments, establishment of 1 dentist as their primary dental care provider). The race/ethnicity variable was recoded to reflect White/non-White categories for analyses, on account of the low representation of any single non-White race or ethnicity. Participants then responded to items related to perceived value of dentistry. These items included the following: “Dental health is important to overall health,” “Dental health is important to me,” “Dentistry is an ‘essential service’ in the community,” and “It is important to visit a dental professional regularly for a dental checkup.” A 7-point Likert scale (*strongly agree* to *strongly disagree*) was used to measure perceptions. These items were scaled together (Cronbach’s alpha = 0.89) to create a composite variable reflective of the perceived value of dentistry. A *strongly agree* response was coded as a 3, *agree* as 2, and *somewhat agree* as 1. All other responses were coded as 0. The items were then summed. The scale was intended to reflect agreement to the statements; therefore, only positive agreement scores were used in the scale, which also prevented having negative values in the analyses.

The next set of questions included items measuring perceptions relating to susceptibility of contracting COVID-19 and dentistry. Again, a 7-point Likert scale (*strongly agree* to *strongly disagree*) was used to estimate agreement with the following statements: “Compared to the rest of the population, my dental professional has a greater risk of transmitting COVID-19,” “Compared to the rest of the population, my dental professional has a greater risk of getting COVID-19,” “I am concerned about contracting COVID-19 from my dental professionals,” “I am concerned about contracting COVID-19 from other patients in my dental office,” “I feel more likely to get COVID-19 in a dental office than in a grocery store,” “I feel more likely to get COVID-19 in a dental office than in a movie theater,” “I feel more likely to get COVID-19 in a dental office than in an airplane,” “I feel more likely to get COVID-19 in a dental office than in the crowd of a concert or athletic event,” and “I feel more likely to get COVID-19 in a dental office than in a hospital where patients with COVID-19 are being treated.” The same coding strategy described previously was used to create a composite variable that measured agreement with statements that reflected perceived susceptibility (Cronbach’s alpha = 0.86). A square root transformation was used to normalize this variable for analyses.

Participants next reported their agreement with statements reflecting attitudes and beliefs (as a single construct) about COVID-19 and dentistry, using the 7-point scale. These items included the following: “Getting COVID-19 is more risky to my overall health than not attending a dental check-up appointment,” “Getting COVID-19 is more risky to my overall health than not attending a dental appointment for restorative work (fillings, crowns, implants, periodontal therapy, root canals, extractions),” “Getting COVID-19 is more risky to my overall health than not attending a dental appointment to address dental pain or infection,” “Social distancing is important to me to prevent the spread of COVID-19,” “I am aware of government recommendations to dental professionals for appropriate dental treatment during the COVID-19 outbreak,” “I trust that my dental office is in compliance with governmental recommendations to dental professionals for appropriate dental treatment during the COVID-19 outbreak,” “I am concerned that visiting my dental provider will cause a shortage in personal protective equipment (masks, gloves) available for healthcare providers fighting COVID-19,” and “The risk of getting COVID-19 in my dental office outweighs the risk of me not getting necessary dental treatment completed.” The same coding strategy described previously was used to construct this composite variable measuring agreement to statements reflecting attitudes and beliefs (Cronbach’s alpha = 0.72). A square root transformation was used to normalize this variable for analyses.

Last, respondents were asked to report their agreement with statements regarding possible events and conditions that would be required for them to feel comfortable returning to regular dental appointments. Statements included the following: “A statement from public health officials that dental offices can operate with minimal risk to patients,” “The government to allow dental offices to operate normally,” “A personal invitation from my dentist inviting me to return,” “Seeing my friends or neighbors return to their dentists for routine dental care,” “The advice of a trusted colleague, friend or family member,” “Having my dental insurance benefits restored,” “Lifting of social distancing requirements,” and “Vaccination available for COVID-19.” The same 7-point Likert scale was used that included options from *strongly agree* to *strongly disagree*.

### Analysis

Data were cleaned and analyzed with STATA 16 (StataCorp). The researchers examined item missingness, means, and distributions. It was determined that item missingness was not widespread, and so no data imputations were needed. Data transformations were done to normalize variables for analyses where needed. Hypothesis testing was conducted with multivariate regression analyses to control for demographic factors that might confound the relationships between independent and dependent variables.

## Results

The average age of respondents in this study was 40 y, and 43.8% were female (Table 1). Nearly half of the sample (46%) reported completion of a 4-y college degree, and over half (55%) indicated an annual household income of at least $50,000 to $59,000. A majority of respondents (84%) stated that they visited the dentist at least annually. A large proportion of respondents (89%) had been to a dentist in the last year, with 81% having chosen 1 dentist to be their primary dental health provider. Most respondents (72%) reported that they brush their teeth twice daily, as compared with 39% saying that they flossed daily (data not shown).

**Table 1:** Participant Demographics (*n* = 449).

Variable	*n*	%	Variable	*n*	%
Gender			Highest education level completed		
Female	198	43.81	Less than high school	1	0.22
Male	250	55.31	High School graduate	60	13.33
Other	4	0 .88	Some college	76	16.89
Race/ethnicity^a^			2-y degree	54	12.00
White/Caucasian	351	77.65	4-y degree	209	46.44
Hispanic/Latino	22	4.87	Master degree	40	8.89
Black or African American	26	5.75	Doctoral degree	10	2.22
American Indian or Alaska Native	1	0.22	Income, $		
Asian	38	8.41	<10,000	11	2.44
Native Hawaiian or Pacific Islander	1	0.22	10,000 to 19,999	25	5.56
Multiracial	12	2.65	20,000 to 29,999	51	11.33
Other	1	0.22	30,000 to 39,999	61	13.56
Current US region of residence			40,000 to 49,999	39	8.67
Midwest	90	19.82	50,000 to 59,000	62	13.78
Northeast	100	22.03	60,000 to 69,999	37	8.22
South	161	35.46	70,000 to 79,999	48	10.67
West	103	22.69	80,000 to 89,999	26	5.78
Current employment status			90,000 to 99,999	12	2.67
Working (paid employee)	315	70.00	100,000 to 149,999	53	11.78
Working (self-employed)	65	14.44	>150,000	25	5.56
Not working^b^	25	5.56	Relationship status		
Not working (looking for work)	9	2.00	Single and not cohabitating	185	41.02
Not working (retired)	21	4.67	Single and cohabitating	37	8.20
Not working (disabled)	3	0.67	Married	193	42.79
Not working (other)	6	1.33	Separated	2	0.44
Not working^c^	6	1.33	Divorced	27	5.99
			Widowed	7	1.55

Age: 40.7 ± 11.2 y (mean ± SD), *n* = 451.

a Race/ethnicity was recoded to reflect White/non-White categories for analyses on account of the low representation of any single non-White race or ethnicity.

b Temporary furlough from a job with no pay or benefits.

c Temporary furlough from a job with partial pay or benefits.

Table 2 lists the mean agreement for each survey question used to create the composite theory variables regarding agreement with perceived susceptibility and attitudes and beliefs of risk associated with COVID-19 and dental appointments. Respondents indicated that contracting COVID-19 from other patients in a dental office posed their greatest risk related to dental care. There was also general agreement that contracting COVID-19 was riskier to overall health than attending a dental checkup or receiving dental restorative work. Last, respondents strongly agreed that social distancing was important to stopping the spread of COVID-19 and that their dental office would be in compliance with governmental COVID-19 recommendations.

**Table 2.** COVID-19 and Dental Appointments Perceived Susceptibility and Attitudes and Beliefs.

	*n*	Mean^a^	SD
Perceived susceptibility to contracting COVID-19 from attending a dental appointment			
Compared to the rest of the population, my dental professional has a greater risk of			
Transmitting COVID-19	447	3.8	1.6
Getting COVID-19	448	3.8	1.7
I am concerned about contracting COVID-19 from			
My dental professionals	447	3.7	1.7
Other patients in my dental office	447	3.3	1.8
I feel more likely to get COVID-19 in a dental office than in			
A grocery store	449	4.3	1.8
A movie theater	448	4.7	1.8
An airplane	447	5.2	1.6
The crowd of a concert or athletic event	447	5.4	1.7
A hospital where patients with COVID-19 are being treated	448	5.7	1.6
Composite perceived susceptibility variable	442	5.5	5.2
Attitudes and beliefs regarding risk of contracting COVID-19 from attending a dental appointment			
Getting COVID-19 is more risky to my overall health than not attending			
A dental check-up appointment	448	2.6	1.5
A dental appointment for restorative work (fillings, crowns, implants, periodontal therapy, root canals, extractions . . . )	449	2.9	1.6
A dental appointment to address dental pain or infection	449	3.9	1.9
Social distancing is important to me to prevent the spread of COVID-19	448	1.8	1.3
I am aware of government recommendations to dental professionals for appropriate dental treatment during the COVID-19 outbreak	446	3.8	1.9
I trust that my dental office is in compliance with governmental recommendations to dental professionals for appropriate dental treatment during the COVID-19 outbreak	448	2.2	1.2
I am concerned that visiting my dental provider will cause a shortage in personal protective equipment (masks, gloves, . . . ) available for healthcare providers fighting COVID-19	447	4.5	1.9
The risk of getting COVID-19 in my dental office outweighs the risk of me not getting necessary dental treatment completed	447	3.6	1.7
Composite attitude and belief variable	443	10.6	5.2

Score, 1 to 7: 1 = strongly agree, 2 = agree, 3 = somewhat agree, 4 = neither agree nor disagree, 5 = somewhat disagree, 6 = agree, 7 = strongly disagree. A square root transformation was used to normalize the composite susceptibility and attitude and belief variables for analyses.

a Mean level of agreement with statement.

Table 3 shows results from a multivariate regression exploring factors associated with increased agreement to statements reflecting perceptions of susceptibility. Respondents who reported greater agreement with the statement indicating that they would likely contract COVID-19 from a dental visit were significantly more likely to have higher agreement to statements about perceptions of susceptibility in dental settings (*P* < 0.001). Increased agreement to statements reflecting increased attitudes and beliefs of risk related to COVID-19 and dentistry were also significantly associated with increased agreement to statements about perceived susceptibility (*P* < 0.001), as was older age (*P* = 0.01). Income was negatively associated with agreement to statements about perceived susceptibility in that higher income was related to decreased perceptions (*P* = 0.03) of susceptibility. Factors associated with increased attitudes and beliefs of risk for contracting COVID-19 from dental visits included increased perceptions of susceptibility (*P* < 0.001), increased perceptions of the value of dentistry (*P* = 0.01), and agreement that COVID-19 is a serious infection (*P* = 0.002; Table 4).

**Table 3.** Regression for Perceived Susceptibility to Contracting COVID-19 from Attending a Dental Appointment (*n* = 432).

Perceived Susceptibility	Coefficient	SE	*t*	*P* > |*t*|	95% CI	
Perceived likelihood of infection	−0.91	0.14	−6.43	0.000	−1.18	−0.63
Perceived value of dentistry	−0.10	0.08	−1.25	0.213	−0.25	0.06
Attitudes and beliefs	0.34	0.05	7.65	0.000	0.26	0.43
Gender	−0.43	0.46	−0.95	0.343	−1.33	0.47
Age	0.05	0.02	2.56	0.011	0.01	0.09
Education	0.01	0.19	0.03	0.977	−0.36	0.37
Race/Ethnicity	−0.02	0.55	−0.03	0.975	−1.10	1.06
Income	−0.17	0.08	−2.12	0.035	−0.32	−0.01

**Table 4.** Regression for Attitudes and Beliefs Regarding Risk of Contracting COVID-19 from Attending a Dental Appointment (*n* = 431).

Attitudes and Beliefs	Coefficient	SE	*t*	*P* > |*t*|	95% CI	
Perceived susceptibility to contracting COVID-19 in dental setting	0.28	0.04	7.27	0.000	0.20	0.36
Perceived value of dentistry	0.32	0.07	4.41	0.010	0.18	0.46
Gender	0.43	0.43	1.02	0.309	−0.40	1.27
Perceive COVID-19 to be serious	1.31	0.14	9.11	0.000	1.03	1.59
Age	−0.02	0.02	−1.18	0.240	−0.06	0.02
Race/ethnicity	0.78	0.51	1.53	0.127	−0.22	1.78
Education	−0.01	0.17	−0.05	0.963	−0.35	0.33
Income	0.10	0.07	1.33	0.183	−0.05	0.24

Respondents indicated agreement that a statement from public health officials regarding minimal risk of dental visits was the event that would give them the greatest comfort returning to a dental office (Table 5). Other events that respondents strongly agreed would help them feel comfortable returning to the dental office included a vaccination for COVID-19, lifting of social distancing requirements, and the government allowing dental offices to operate normally. Respondents reported that seeing friends or neighbors return for dental care and receiving advice of trusted colleagues, friends, or family members as events that gave them the least confidence in returning to a dental office.

**Table 5.** Level of Agreement with Events That Need to Occur for Patients to Feel Comfortable Returning to the Dental Office.

Event	*n*	Mean	SD
Vaccination available for COVID-19	446	2.7	1.8
Lifting of social distancing requirements	447	2.8	1.6
Having my dental insurance benefits restored	448	3.3	1.6
The advice of a trusted colleague, friend, or family member	448	3.8	1.5
Seeing my friends or neighbors return to their dentists for routine dental care	445	4.1	1.5
A personal invitation from my dentist inviting me to return	445	3.7	1.6
A statement from public health officials that dental offices can operate with minimal risk to patients	447	2.6	1.5
The government to allow dental offices to operate normally	448	2.9	1.5

Score, 1 to 7: 1 = strongly agree, 2 = agree, 3 = somewhat agree, 4 = neither agree nor disagree, 5 = somewhat disagree, 6 = agree, 7 = strongly disagree.

## Discussion

The purpose of this study was to explore the perceptions of susceptibility to contracting COVID-19 and the related attitudes and beliefs of dental patients regarding professional dental care visits during the COVID-19 pandemic. A secondary purpose of this study was to determine the conditions and events that may influence when dental patients return to pre–COVID-19 dental appointment regimens. This study found that age and income were important predictors of susceptibility for contracting COVID-19 in a dental setting. Furthermore, results indicated that attitudes and beliefs of risk regarding COVID-19 were influenced by increased perceptions of susceptibility and the belief that COVID-19 is a serious infection. Last, results indicated that assurance from public health officials of the safety to return for routine dental care was the largest factor contributing to when a patient would seek care again.

As respondents increased in age, perception of susceptibility to contracting COVID-19 was higher. This is consistent with messaging from the CDC (2020c) that risk for greater morbidity and mortality is higher among older individuals with COVID-19. Hospitalizations are also highest in adults ≥65 y (Garg et al. 2020). In contrast to age, there was an inverse relationship between income and perceived susceptibility. Those with lower incomes perceived themselves as being more susceptible to contracting COVID-19. Researchers have long demonstrated that persons of high economic status are likely to be healthier than persons of low socioeconomic standing (Semyonov et al. 2013). It is well established in the literature that poverty and contracting infectious diseases are related (Bonds et al. 2009). In the early days of this pandemic, the relationship between income levels and rates of infection with COVID-19 was unclear. However, researchers have reported that low-income households are now more likely to have conditions associated with increased risk of illness from COVID-19 relative to those who are higher income. Structural disparities such as wealth, medical insurance, and income volatility compound this increased risk (Raifman and Raifman 2020). An additional challenge to the psychological barrier of increased perceived susceptibility to contracting COVID-19 in dental offices is that significant socioeconomic barriers to dental care already exist for the aged and the poor. Historically, these 2 vulnerable populations have poor professional dental utilization and a higher incidence of unmet dental needs (Raphael 2017; Zhou et al. 2017). As dental providers work to provide a safe environment for patients as they begin to return for care, these findings suggest that it will be important to work closely with aging and low-income populations to ensure that worries regarding susceptibility are being addressed. One area for immediate attention is for dental professionals to communicate that they are taking every possible precaution in their offices to prevent infection from provider to patient and from patient to patient. These were among the most concerning scenarios to participants in this study.

Results from an analysis of attitudes and beliefs demonstrated an agreement with a cautious approach to interacting with dental professionals to protect the patient and provider. Increased perceived susceptibility, increased perception of the severity of the disease, and high perceived value or esteem for oral health and dental services were all related to a cautious approach toward attending dental appointments. It is interesting that those who value oral health would take a more cautious approach toward attending dental appointments. On initial inspection, these 2 attitudes may seem contrary to each other. According to respondents, even though dental health is highly important, it is not more important than keeping safe from contracting COVID-19. It is understandable then that patients who reported a high perceived value or esteem for oral health and dental services also reported a higher level of attitudes and beliefs of caution. This is in spite of the findings from other research showing statistically significant relationships among oral health, energy levels, work limitation, depression, and appetite as well as physical, mental, and general health (Hung et al. 2019). Messaging by dental professionals based on this finding may benefit from a concerted effort to reinforce the importance of maintaining good oral health in times of suspended routine checkups. Clinicians may assume an important role by communicating with patients and reminding them of the value of routine dental services to their overall health and that these important services will resume in their fullness as soon as possible because providers value the overall health of their patients. Most patients in this study believed that their dental office is complying with government guidelines, so it is likely that patients also view their dental professionals as credible sources of health information. For these reasons, communicated efforts by dental practitioners to promote preventive dental visits practices could be beneficial.

The goal of dental public health and dental primary care practitioners during this pandemic is to return the population to good oral health habits while not increasing the spread of COVID-19. Patients and providers need confidence that a return to routine dental care will provide a net benefit to the population. Respondents in this study reported that they would feel most comfortable returning to dental offices following actions from public health experts, including vaccine development, easing of social distancing requirements, and appropriate recommendations. Less important to respondents are the actions or advice of trusted colleagues, family, friends, and neighbors. This is in contrast to many other less serious daily life events, which are highly influenced by peer groups and intimate social units (Petosa and Smith 2014). This observation reemphasizes the gravity of the COVID-19 pandemic and the serious nature of perceptions and attitudes that individuals have toward the risk of contracting COVID-19. It also speaks to the unique opportunity for the community of dental professionals to partner with leading public health agencies to ensure that dentistry remains visible in messaging efforts as it relates to reopening of states and cities.

The findings from this study should be interpreted in the context of its limitations. First, the study sample, recruited through MTurk, was quite homogeneous, with most respondents reporting to be White and having a high annual income. Whereas this may seem to be an anomaly, it is a consistent demographic composition for MTurk research as reported elsewhere (Shapiro et al. 2013). In this study, MTurk provided a population to survey that has been shown to be highly attentive (Hauser and Schwarz 2016) and accessible with a short turnaround and in a time of decreased opportunity for researchers to interact with potential respondents. Second, this survey was made available in early May 2020. During this time, a general flattening of the epidemic curve was being reported, and some participants, feeling more confident about the improving direction of the pandemic, may have demonstrated desirability bias and responded in a way that reflected more favorably upon the slowing of the pandemic. Further cross-sectional surveys at regular intervals would reflect the reality of the pandemic in its entirety more accurately. Third, this survey relied completely on self-report data. People are not necessarily good predictors of their own future behavior, so the data may not reflect actual future outcomes, such as treatment-seeking patterns. Last, survey items were constructed to address the purpose of the current study. There were no previous studies of perceptions of COVID-19 and dentistry, so comparisons of the findings in this study with those in previous research may be limited. In response to the lack of existing items, the HBM and pilot testing were used to increase the validity of the survey items and instrument that were created.

## Conclusion

It is important for public health policies to be evidence based. The lack of published research on COVID-19 and dental professionals is to be expected, yet it is a challenge for clinicians and policy makers wishing to make empirically based policies. This study provides early data to identify patient perceptions of risk susceptibility and attitudes toward COVID-19 in a professional dental setting. This study also provides information about the conditions and events that may influence patients’ confidence to return to regular dental visits. Government and public health agencies can play an important role in alleviating concerns and instilling confidence that dental settings are safe. With this information from the public, dental professionals and public health agencies can work together to share messaging that will consistently inform the public regarding the safety of returning to professional dental care as it relates to the reopening of states and cities. Such messaging may need to target the most susceptible populations and work to address fears of contracting the virus.

## Author Contributions

R.C. Moffat, contributed to conception, design, and data analysis, drafted and critically revised the manuscript; C.T. Yentes, J.H. West, contributed to data conception, design, and data analysis, critically revised the manuscript; B.T. Crookston, contributed to data analysis, critically revised the manuscript. All authors gave final approval and agree to be accountable for all aspects of the work.

## References

[bibr1-2380084420969116] American Dental Association. 2020 ADA recommending dentists postpone elective procedures. Chicago (IL): American Dental Association; [accessed 2020 5 26]. https://www.ada.org/en/publications/ada-news/2020-archive/march/ada-recommending-dentists-postpone-elective-procedures

[bibr2-2380084420969116] Bonds MH Keenan DC Rohani P Sachs JD 2009 Poverty trap formed by the ecology of infectious diseases. Proc R Soc B. 277(1685):1185–1192. 10.1098/rspb.2009.1778 PMC2842808 20007179

[bibr3-2380084420969116] Centers for Disease Control and Prevention. 2020a. SARS-CoV-2 and potential airborne transmission. Atlanta (GA): Centers for Disease Control and Prevention; [accessed 2020 Oct 15]. https://www.cdc.gov/coronavirus/2019-ncov/more/scientific-brief-sars-cov-2.html

[bibr4-2380084420969116] Centers for Disease Control and Prevention. 2020b. Information for healthcare professionals about coronavirus (COVID-19). Atlanta (GA): Centers for Disease Control and Prevention; [accessed 2020 5 26]. https://www.cdc.gov/coronavirus/2019-ncov/hcp/index.html

[bibr5-2380084420969116] Centers for Disease Control and Prevention. 2020c. Coronavirus disease - older adults. Atlanta (GA): Centers for Disease Control and Prevention; [accessed 2020 Oct 15]. https://www.cdc.gov/coronavirus/2019-ncov/need-extra-precautions/older-adults.html

[bibr6-2380084420969116] Champion VL Skinner CS 2008 Health behavior and health education: theory, research, and practice. 4th ed. San Francisco (CA): Jossey-Bass p. 45–65.

[bibr7-2380084420969116] Chen X Shang Y Yao S Liu R Liu H. 2020 Perioperative care provider’s considerations in managing patients with the COVID-19 infections. Transl Perioper Pain Med. 7(2):216–224.

[bibr8-2380084420969116] Chustecka Z. 2020 More than 60 doctors in Italy have died in COVID-19 pandemic. Medscape Medical News [updated 2020 3 31; accessed 2020 5 26]. https://www.medscape.com/viewarticle/927753

[bibr9-2380084420969116] Garg S Kim L Whitaker M O’Halloran A Cummings C Holstein R Prill M Chai SJ Kirley PD Alden NB, et al 2020 Hospitalization rates and characteristics of patients hospitalized with laboratory-confirmed coronavirus disease 2019—COVID-NET, 14 states, 3 1–30, 2020 MMWR Morb Mortal Wkly Rep. 69(15):458–464. Published erratum. 10.15585/mmwr.mm6915e3 PMC7755063 32298251

[bibr10-2380084420969116] Hauser DJ Schwarz N. 2016 Attentive Turkers: MTurk participants perform better on online attention checks than do subject pool participants. Behav Res Methods. 48(1):400-407. 25761395 10.3758/s13428-015-0578-z

[bibr11-2380084420969116] Hung M Moffat R Gill G Lauren E Ruiz-Negrón B Rosales MN Licari FW 2019 Oral health as a gateway to overall health and well-being: surveillance of the geriatric population in the United States. Spec Care Dent. 39(4):354–361. 10.1111/scd.12385 31087569

[bibr12-2380084420969116] Johns Hopkins University and Medicine. 2020 Corona Virus Resource Center. Baltimore (MD): Johns Hopkins University and Medicine; [accessed 2020 5 26]. https://coronavirus.jhu.edu/map.html

[bibr13-2380084420969116] Meng L Hua F Bian Z. 2020 Coronavirus disease 2019 (COVID-19): emerging and future challenges for dental and oral medicine. J Dent Res. 99(5):481–487. 32162995 10.1177/0022034520914246 PMC7140973

[bibr14-2380084420969116] Petosa RL Smith LH 2014 Peer mentoring for health behavior change: a systematic review. Am J Health Educ. 45(6):351–357.

[bibr15-2380084420969116] Raifman MA Raifman JR. 2020 Disparities in the population at risk of severe illness from COVID-19 by race/ethnicity and income. Am J Prev Med. 59(1):137–139. 32430225 10.1016/j.amepre.2020.04.003 PMC7183932

[bibr16-2380084420969116] Raphael C. 2017 Oral health and aging. Am Pre J Public Health. 107(S1):S44–S45. 10.2105/AJPH.2017.303835 PMC5497890 28661797

[bibr17-2380084420969116] Semyonov M Lewin-Epstein N Maskileyson D. 2013 Where wealth matters more for health: the wealth-health gradient in 16 countries. Soc Sci Med. 81:10–17. 23422055 10.1016/j.socscimed.2013.01.010

[bibr18-2380084420969116] Shapiro DN Chandler J Mueller PA 2013 Using mechanical Turk to study clinical populations. Clin Psychol Sci. 1(2):213–220.

[bibr19-2380084420969116] Zhou JY Elyasi M Amin M. 2017 Associations among dental insurance, dental visits, and unmet needs of US children. J Am Dent Assoc. 148(2):92–99. 28129806 10.1016/j.adaj.2016.11.013

